# DIFFERENCE IN TIME SPENT CAREGIVING BETWEEN CAREGIVERS OF IMMIGRANT AND NONIMMIGRANT OLDER ADULTS

**DOI:** 10.1093/geroni/igad104.1623

**Published:** 2023-12-21

**Authors:** Zainab Suntai, Joana Okine

**Affiliations:** Baylor University, Waco, Texas, United States; University of Alabama, Tuscaloosa, Alabama, United States

## Abstract

Each year, the number of informal caregivers in the United States continues to rise, in conjunction with the increasing aging population. Concurrently, the number of immigrants in the United States had progressively increased over the past few decades, leading to an intersection of three pivotal issues: caregiving, aging, and immigration. While there are several studies targeting caregiving in the United States, fewer studies have aimed to understand the experiences of caregivers of older immigrants. This study examined differences in time spent caregiving between caregivers of older immigrants compared to caregivers of older non-immigrants. Data were taken from Round 11 (2022) of the National Study of Caregiving, a nationally representative study of informal caregivers caring for an older adult with functional limitations. Chi-square tests were used to test bivariate associations, and a multivariate logistic regression model was run to determine the relationship between and older adult’s immigration status and time spent caregiving by their caregiver. Results showed that caregivers of older immigrants were significantly more likely to report that they provided care every day, compared to caregivers of non-immigrants. These results point to potential cultural implications for immigrant families, who are generally less likely to use formal caregiving avenues such as nursing homes, and less likely to use additional services such as home health aides. Implications for researchers, policymakers, and practitioners are discussed.

